# Motivational interviewing for substance use among adults in low- and middle-income countries: A systematic review

**DOI:** 10.4102/sajpsychiatry.v32i0.2555

**Published:** 2026-05-13

**Authors:** Amy S. Adams, Taryn Williams, Dan J. Stein, Goodman Sibeko, Steven Shoptaw, Stephen Rollnick

**Affiliations:** 1 Department of Psychiatry and Mental Health, Faculty of Health Sciences, University of Cape Town, Cape Town, South Africa; 2 Department of Family Medicine, Faculty of Health Sciences, University of California Los Angeles, Los Angeles, United States; 3 School of Medicine, Faculty of Health Sciences, Cardiff University, Wales, United Kingdom

**Keywords:** motivational interviewing, motivational interviewing-informed interventions, substance use disorders, LMICs, addiction, harmful substance use, cognitive-behavioural therapy, behavioural activation, imaginal desensitisation

## Abstract

**Background:**

Harmful substance use (HSU) poses a significant global health challenge, impacting social, economic, and healthcare systems. Low- and middle-income countries (LMICs) are particularly burdened as a result of limited infrastructure for managing substance-related issues. While evidence-based brief interventions like motivational interviewing (MI) are widely used in high-income countries, research on their effectiveness in LMICs is limited.

**Aim:**

This systematic review aims to evaluate studies employing MI as part of interventions for adults with HSU in LMIC settings.

**Setting:**

This review focused on studies within low- and middle-income countries which include settings characterised by constrained mental health resources, high treatment gaps for substance use disorders (SUDs), and diverse service delivery contexts.

**Methods:**

Following Cochrane and PRISMA guidelines, we searched PubMed, EBSCOhost, Web of Science, Cochrane Library, clinicaltrials.gov and GSK Clinical Study Register for publications from 2010 to 2022. A narrative analysis was conducted, and the Joanna Briggs Institute Critical Appraisal Tools assessed study quality.

**Results:**

Out of 518 publications, 11 studies from five countries met our inclusion criteria. Most were randomised controlled trials, with some using qualitative or mixed methods. The interventions targeted alcohol, opioids, and other drugs. Different strategies were used, including MI-blended with other interventions (such as cognitive-behavioural therapy, behavioural activation, and imaginal desensitisation) or using MI-informed approaches.

**Conclusion:**

Overall, MI-informed interventions were shown to improve substance use outcomes in six of the 11 studies, with a significant reduction in substance use. However, further high-quality research with larger samples and rigorous designs is needed to strengthen the evidence base and address potential sources of bias.

**Contribution:**

Findings suggest that MI-informed approaches, as a brief intervention, can improve substance use outcomes in resource-constrained environments within LMICs.

## Introduction

Harmful substance use (HSU) is a major challenge globally with far-reaching detrimental effects on social, economic, and health systems.^1^ According to the World Health Organization (WHO), in 2019, more than 180 000 deaths were directly linked to drug use disorders.^2^ Recent WHO Global Health Estimates and Global Burden of Disease analyses indicate that, while cardiometabolic and tobacco-related exposures remain major global risk factors, drug use disorders are still an important contributor to morbidity and mortality – particularly in younger age groups and certain regions – and were ranked among the top 20 causes of Disability-Adjusted Life Years (DALYs) for people aged approximately 10–49 years in 2019.^3^ According to the WHO’s 2024 Global Status Report, harmful alcohol consumption in 2019 accounted for an estimated 2.6 million deaths globally (≈ 4.7% of all deaths) and about 115.9m DALYs (≈ 4.6% of all DALYs).^3^ A report by the United Nations Office on Drugs and Crime^4^ provides global estimates of substance use prevalence according to the substance. According to their report, the best estimate for global prevalence of cannabis use is at 4.12%, opioids 1.21%, cocaine 0.42%, amphetamines and prescription stimulants 0.68% and ecstasy 0.39%.^4^ In low- and middle-income countries (LMICs), the best estimate for the prevalence of cannabis use ranges from 2.77% to 12.00%, opioids from 1.00% to 3.20%, cocaine from 0.04% to 2.70%, amphetamines and prescription stimulants from 0.28% to 1.26% and ecstasy from 0.23% to 2.84%.^4^

Harmful substance use is an umbrella term used to refer to licit and illicit substances, including alcohol, nicotine, and drugs such as heroin, cocaine, cannabis, amphetamines, methamphetamines, and pharmaceutical drugs such as opioids and benzodiazepines.^5^ Harmful substance use can therefore be defined as the use of substances harming an individual, which may include problems in the areas of health, social life, finances, emotional problems, and possibly legal problems.^5^ The social burden of HSU is significant and is commonly associated with health concerns, impairment in education, and an increasing tendency towards crime and unemployment.^6^ This burden weighs more heavily on developing countries as they do not have the infrastructure for managing the consequences of substance use.^7^ The World Mental Health Survey showed that 76% to 85% of the patients with severe mental illnesses (e.g. substance use disorders [SUDs], anxiety or mood disorders) in LMICs received no treatment for their conditions over 12 months.^8^

In high-income countries, several evidence-based brief intervention models are used for HSU, including motivational interviewing (MI) alone or with cognitive-behavioural therapy (CBT), stress management, problem-solving, case management and community contingency therapy.^9,10^ However, fewer studies have been conducted in low-resource settings.^11^ Multiple factors make delivering effective treatment for HSU challenging in LMICs.^12^ These challenges include the large geographical spread of health facilities, poorly trained treatment providers to deliver the interventions, stigmatising attitudes by providers towards those presenting with HSU, governance that accords low priority for HSU, heavy staff workload,^13^ poorly resourced health facilities, and minimal funding to employ adequate staff among other factors; all contribute towards insufficient services offered to patients presenting with HSU.^11^

Motivational interviewing is a collaborative, client-centred counselling approach focused on strengthening an individual’s motivation and commitment to change.^14^ It is not a single uniform intervention, but encompasses several formats that differ in dose, structure, and application, including *full or standard MI*, typically delivered over multiple sessions, *brief MI*, which is shorter and adapted for time-limited settings, and *MI-informed or MI-consistent approaches*, in which practitioners apply MI principles without delivering a full protocol.^14,15,16^ These variations are widely recognised in both foundational MI literature and empirical reviews and are important to consider when comparing outcomes across studies.^17,18^

The effectiveness of MI as an intervention for substance use in high income countries (HICs), in its various formats as highlighted above, is mainly positive, but has also produced mixed results. Studies from HICs demonstrate that standard MI improves outcomes for non-injection drug users^9^; brief MI is effective for patients presenting with AUDs^19^; and MI-consistent interventions have been shown to improve treatment response^20^ and are useful when used for co-occurring alcohol use and depression^21,22^ and have been found effective in the treatment of first-episode psychosis and SUDs in young adults.^23^ However, in a meta-analysis by Wang and colleagues,^24^ the use of pure MI to treat psychosis and SUDs was found to be varied with relatively modest positive outcomes. Moyers and Houck^25^ point out that MI combined with other treatments may at times be challenging, as the difference in approaches may be contradictory. They further state that combined treatment, although effective, may not always be seamless or without difficulty.^25^

The evidence of the effectiveness of MI for SUD treatment in LMICs is substantially less. In a meta-analysis on psychosocial interventions (including three MI-informed and/or MI-consistent interventions) for reducing alcohol consumption in sub-Saharan Africa, the outcomes were mixed.^26^ The meta-analyses concentrating on Alcohol Use Disorders Identification Test (AUDIT) scores discovered no statistically significant distinctions between the intervention and the comparison group at intervals of 2–3 months, 6 months, or 12 months following the intervention. However, reviewing trials related to alcohol abstinence revealed a positive impact of psychosocial interventions compared to the impact on the comparison group at the 3–6-month mark post-intervention, with sustained effect at longer-term follow-up periods (12–60 months).^26^

Outcomes of intervention research in HICs may not yield similar results in LMICs even after being adapted to the local context in low-income populations.^27^ Given the various challenges faced in LMICs, of utmost importance is to identify and implement brief, innovatively delivered, cost-effective solutions that empower providers in primary care settings to treat HSU effectively.^11^ This review, therefore, aims to determine whether HSU interventions incorporating MI are effective with adults in LMICs. We further aim to highlight any limitations within the literature and offer opportunities for future research. This information will be of value to relevant stakeholders working in the field of addiction in LMICs to help make treatment and policy decisions within their given context.

## Research methods and design

Methods were pre-specified and documented in a protocol (PROSPERO–CRD42022329196). The inclusion and exclusion criteria are discussed in the next section.

### Inclusion criteria

Studies were included in this review if they met the following criteria:

- Studies that delivered MI in any of the following formats:
  ■ Full and/or standard MI, defined as a multi-session, fidelity-adherent MI intervention delivered according to Miller and Rollnick’s principles and processes.
  ■ Brief MI, defined as a single-session or short-series MI intervention (typically 5–30 min) delivered using core MI strategies.
  ■ Motivational interviewing-informed and/or MI-consistent interventions, defined as programmes incorporating selected MI principles or techniques without delivering a full MI protocol, often combined with a co-intervention such as CBT and problem-solving therapy (PST), among others.
- We did not restrict eligibility based on study design.
- Included adults aged 18 years and older.
- Included participants presenting with HSU.
- A diagnosis of an SUD as defined by any operationally defined criteria (e.g. DSM-5, ICD-11, or similar).
- Included participants presenting with or without comorbid mental disorders secondary to SUD (e.g. mood disorders, anxiety disorders).
- Conducted in LMICs and settings.
- Only studies published in English were included, and no translations were required.

### Exclusion criteria

Studies were excluded based on the following criteria:

- Those with participants with major severe mental illness (e.g. schizophrenia, bipolar I disorder), as these conditions present an additional challenge to the use of MI as a treatment approach.
- Those examining healthy populations, children, and adolescents.
- Those in high-income countries (HICs) and settings.

### Search and data abstraction

The databases searched were PubMed (which includes Medline), EBSCOhost (which includes PsycINFO), Web of Science and The Cochrane Library. Both clinical trial registries (clinicaltrials.gov and the GSK Clinical Study Register) were searched for records published between 2010 and 2022.

The keywords used were all possible combinations of the following words and phrases: ‘motivational interviewing’, ‘mi’, ‘motivational intervention’, ‘addiction’, ‘substance use’, ‘alcohol use’, ‘drug use’, ‘tobacco use’, ‘harmful substance use’, ‘adults’, ‘qualitative research’, ‘quantitative research’, ‘low- and middle-income countries’, ‘developing countries’ and ‘developing nations’.

The lead reviewer (Amy S. Adams) systematically evaluated the titles, abstracts, and keywords associated with each article to determine their fulfilment of the inclusion or exclusion criteria. Of the 518 articles found, 16 were included and then reviewed more closely by two authors (Amy S. Adams and Taryn Williams), and a further nine articles were eliminated. Any confusion or ambiguity about suitability was discussed with the second author (Taryn Williams) and resolved between the two authors. The reference lists of the remaining seven articles were screened, and a further four articles were deemed suitable for inclusion. Therefore, a total of 11 articles were considered suitable for inclusion in this review. The PRISMA guidelines were followed, and the PRISMA search flow diagram was used for this review.^28^

After the articles were identified, Amy S. Adams and Taryn Williams independently extracted the following data: (1) description of the trials, (2) characteristics of participants, (3) characteristics of the intervention, including its duration, (4) outcome measures employed (primary and secondary), and (5) a summary of continuous and dichotomous data. Additional information was also extracted, such as the total dropouts per group. A narrative analysis was then done.

#### Assessment of the quality of evidence

Two reviewers (Amy S. Adams and Taryn Williams) independently assessed the methodological quality of included studies, using the Joanna Briggs Institute (JBI) Critical Appraisal Tool for randomised controlled trials (RCTs),^29^ qualitative research^30^ and analytical cross-sectional studies.^31^ The Joanna Briggs checklist for RCTs includes 13 items covering two domains, including *internal validity* (Was true randomisation used for assignment of participants to treatment groups? Was allocation to treatment groups concealed? etc.) and *statistical conclusion validity* (Were participants analysed in the groups to which they were randomised? Was appropriate statistical analysis used? etc.).^29^ This appraisal for quantitative evidence is to determine the extent to which a study has addressed the possibility of bias in the area of design, conduct and analysis.^29^ The JBI Critical Appraisal Tool for qualitative research includes 10 items and considers specific aspects of the study, such as congruity between the stated philosophical perspective and the research methodology, representation of participants and their voices, and the relationship of conclusions to analysis or interpretation of the data.^30^ The JBI Critical Appraisal Tool for analytical cross-sectional studies includes eight items, including whether inclusion criteria were clearly defined, whether confounding variables were identified, and whether outcomes were measured validly and reliably, among others.^31^

Each study was critically appraised by using the relevant appraisal tool, and based on the analyses, the reviewers had the option to include, exclude, or seek further information, with any disagreement being resolved through discussion between the two reviewers. After an independent review, all 11 selected studies were included.

#### Risk of bias

The risk of bias was assessed for studies with applicable designs. The Cochrane Collaboration ‘Risk of Bias’ tool was used, and the following six domains were considered: sequence generation, allocation concealment, blinding of participants and personnel, blinding of outcome assessors, incomplete outcome data and selective outcome reporting. Additionally, other sources of bias (such as selective reporting and inadequate handling of missing data) were also identified and reported on. A judgement on the risk of bias was also made for each of the six domains mentioned above, based on the following three categories: high risk of bias, low risk of bias and unclear risk of bias.^32^ These aspects were covered in the RCT JBI checklist and helped in making final inclusion decisions.

### Ethical considerations

Ethical approval was not required as the study did not involve human participants or identifiable personal information.

## Results

**FIGURE 1:**
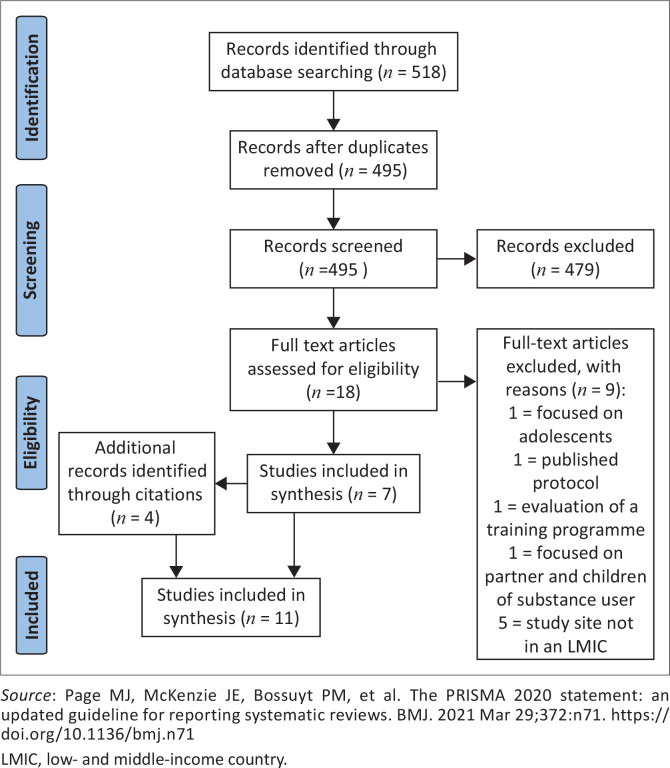
Search flow diagram.

Figure 1 shows the article selection process for this review, according to the PRISMA guidelines. The initial searches yielded 518 records, and 495 remained after removing duplicates. After screening titles and abstracts, 18 were eligible for full review. Full texts were then reviewed, and nine did not meet the inclusion criteria (one article focused on adolescents and not adults; one was a published protocol; one was an evaluation of a training programme; the study sample in one article was the partner and children of the substance-using patient rather than the patient himself; and all others were excluded because the study sites were not based in an LMIC). Examining the reference lists from the remaining seven articles identified four additional studies.^33,34,35,36^ Thus, 11 studies were included in this review.^33,34,35,36,37,38,39,40,41,42,43^

### Trial characteristics

#### Location or setting

As presented in Table 1, four studies were conducted in South Africa,^35,39,42,43^ two in Kenya,^33,37^ one in Uganda,^36^ and two in India.^40,41^ Study settings included primary healthcare centres,^39,40,41^ emergency centres,^42,43^ drop-in centres,^33^ community-based urban and/or peri-urban settings,^37^ specialist clinics^34,36,38^ and rural settings.^35^

**TABLE 1:** Characteristics of included studies.

Author and year	Country	Intervention	Design	Participants	Sample	Mean age	Males (%)	Key finding/s of outcomes
Madhombiro et al., 2019^34^	Zimbabwe (LIC)	MI-CBT: 4 sessions, 30–60 min per session.	RCT feasibility study	AUDIT scores: Men ≥ 7 Women ≥ 6	*N* = 40 Dropouts (*n* = 9)	39.5	57.5	Significant difference in use over time (*p* < 0.001). No significant difference in the magnitude of change, functioning, and QOL. No significant change over time.
Madhombiro et al., 2020^38^	Zimbabwe (LIC)	MI-CBT: 8–10 sessions, 45–60 min per session.	Cluster RCT	On ARVs for ≥ 3 months. AUDIT scores: Men ≥ 7 Women ≥ 6	*N* = 234 Dropouts (*n* = 77)	43.3	78.6	Significant difference in AUDIT score at 6 months post-intervention (*p* < 0.001).
Giusto et al., 2022^37^	Kenya (MIC)	LEAD: BA blended with MI, 5 sessions, 60–90 min per session.	Qualitative	AUDIT score between 8 and 20. Child between the ages of 8 and 17 years	*N* = 9 Dropouts (*n* = 3)	39	100	Reduced alcohol use. Improved: mood, interactions at home, feeling of hope, peace in the home, and money saving.
Marks et al., 2020^39^	South Africa (MIC)	MI-informed milieux therapy (all staff interactions creating an accepting and positive treatment environment) promotes social cohesion and elements of restorative justice.	Qualitative	≥ 12 months of heroin use. ASSIST score: ≥ 27. Recent use of opioids.	*N* = 54 Dropouts (24%)	28	96	High retention rate at 74% and harm reduction. Achieved social cohesion, successfully promoted restorative justice with participants feeling heard and respected, feeling a sense of belonging, and feeling supported.
Nadkarni, 2015^40^	India (MIC)	CAP (MI-informed): 1 to 4 sessions, 30–45 min per session versus EUC comprising mhGAP.	Pilot RCT	AUD or AUDIT score of ≥ 19.	*N* = 53 Dropouts (75.6%)	41.4	100	Reduced alcohol use in the past 2 weeks, but no statistically significant difference between the two treatment groups. Nonsignificant reductions in outcomes between treatment completers and those who dropped out with regard to mean AUDIT (8.6 vs. 12.9, *p* = 0.3), mean alcohol consumed in the past 2 weeks (272.5 g vs. 368.4 g, *p* = 0.6) and mean short inventory of problems (SIP) score (6.4 vs. 8, *p* = 0.7).
Nadkarni, 2019^41^	India (MIC)	CAP (MI-informed): 1 to 4 sessions, 30–45 min per session versus EUC comprising mhGAP.	Exploratory RCT	Attending PHC. AUDIT score of ≥ 20.	*N* = 135 Dropouts (*n* = 23)	43.3 (CAP) 39.7 (EUC)	100	The difference between the arms was not significant for remission at (1) proportion with remission at 3 months (27.1% vs. 14.5%, *p* = 0.18) and 12 months (31.0% vs. 18.5%, *p* = 0.19); (2) proportion of participants reporting no alcohol consumption in the past 14 days at 3 months (35.6% vs. 30.7%, *p* = 0.57) and 12 months (34.5% vs. 29.6%, *p* = 0.57); and (3) consumption among those who reported any drinking in this period at 3 months (58.9 g vs. 59.2 g, *p* = 0.70) and 12 months (45.2 g vs. 60.4 g, *p* = 0.77). For the ‘worst-case scenario’ sensitivity analysis, the difference between the two arms was not significant for the proportion with remission at 3 months (23.2% vs. 13.6%, *p* = 0.29) and 12 months (26.1% vs. 15.2%, *p* = 0.22).
Van der Westhuizen et al., 2021^43^	South Africa (MIC)	One ASSIST-linked MI-informed session followed by two sessions of PST.	Mixed methods	Attending an acute visit at EC. ASSIST scores: ≥ 6 for alcohol, ≥ 1 for drugs.	*N* = 4847 Dropouts = missing information.	33	70	59% of patients reported no alcohol use days at the 3-month follow-up, compared to 11% at screening (*p* = 0.003). Median alcohol scores dropped significantly from the acute EC visit to the 3-month follow-up. The number of drug use days in the previous 2 weeks decreased significantly from screening to follow-up (*p* < 0.001). At the three-month follow-up, 75% of patients using drugs reported no drug use days in the preceding 2 weeks. ASSIST scores for patients using cannabis and methamphetamine decreased.
Wandera et al., 2017^36^	Uganda (MIC)	Positive prevention and advice plus a single brief MI-informed alcohol counselling session lasting 20–30 min.	RCT	Continued care at the AIDC for the next 6 months. AUDIT-C score of ≥ 3.	*N* = 337 Dropouts (*n* = 57)	39	65.6	Overall reduction in mean AUDIT-C score at 6-month follow-up: 6.4 to 3.4 (*p* < 0.0001) in the control arm and 6.8 to 3.9 in the intervention arm (*p* = 0.001). Mean AUDIT-C score at 3 months was significantly lower in the control arm compared to that in the intervention arm (3.5 vs. 4.3, *p* = 0.034). No difference in mean scores between groups at 6 months (3.4 in the control group vs. 3.9 in the intervention group, *p* = 0.141). The proportion of participants with mean AUDIT-C scores ≥ 3 was significantly lower in the control group compared to that in the interventional arm at 3 months (58.5% in the control group vs. 71.5% in the interventional group, *p* = 0.019) and at 6 months (57.2% in the control group vs. 70.2% in the interventional group, *p* = 0.024).
Sorsdahl et al., 2021^42^	South Africa (MIC)	IDMI: 6 sessions, 20 min per session.	RCT feasibility trial	MAUD. ≥ grade 9 level of education.	*N* = 60 Dropouts (*n* = 35)	31.0	53.3	Greater reductions in frequency of methamphetamine use in the treatment group. Statistically significant time by treatment group interaction at both 6 weeks (*p* < 0.01) and 3 months (*p* < 0.01). Consistent with treatment completers at the 3-month follow-up at 6 weeks (*p* < 0.01) and 3 months post-intervention (*p* < 0.01).
L’Engle et al., 2014^33^	Kenya (MIC)	WHO Brief Intervention for Alcohol Use – MI-informed, 6 sessions, 20 min per session.	RCT	AUDIT scores between 7 and 19. FSWs associated with APHIA.	*N* = 818 Dropouts (intervention: 9%; control: 7%)	27.5	0	Significant difference in reduction between the intervention and control group on reduced drinking in the last 30 days at 6-month and 12-month follow-up visits, frequency of drinking alcohol, overall binge-drinking, binge-drinking with paying clients, and binge-drinking with nonpaying partners (*p* < 0.0001).
Rendall-Mkosi et al., 2013^35^	South Africa (MIC)	5-session MI intervention.	RCT	Women at high risk of alcohol-affected pregnancy.	*N* = 165 Dropouts (intervention: 73.39%; control: 77.11%)	29.8	0	Significant difference in the decline of women at risk for an AEP in the MI group (50.82%) versus the control group (28.12%) at 12 months (*p* = 0.009), 50% reduction in the MI group and a 24.59% reduction in the control group (*p* = 0.004) at the 3-month follow-up.

Note: Please see the full reference list of this article Adams AS, Williams T, Stein DJ, Sibeko G, Shoptaw S, Rollnick S. Motivational interviewing for substance use among adults in low- and middle-income countries: A systematic review. S Afr J Psychiat. 2026;32(0), a2555. https://doi.org/10.4102/sajpsychiatry.v32i0.2555 for more information.

AIDC, Adult Infectious Diseases Institute Clinic; AEP, alcohol-exposed pregnancy; ASSIST, alcohol smoking and substance involvement screening test; APHIA, AIDS, Population, Health, and Integrated Assistance II project; AUD, alcohol use disorder; AUDIT, alcohol use disorder identification test; AUDIT-C, ALCOHOL use disorder identification test alcohol consumption questions; BA, behavioural activation; CAP, counselling for alcohol problems; EC, emergency centre; EUC, enhanced usual care; FSW, female sex worker; IDMI, imaginal desensitisation plus motivational interviewing; LEAD, Learn, Engage, Act, Dedicate; LIC, low-income country; MA, methamphetamine; MAUD, methamphetamine use disorder; MIC, middle-income country; MI-CBT, motivational interviewing – cognitive-behavioural therapy; mhGAP, mental health gap action programme; PHC, primary healthcare centre; PST, problem-solving therapy; QOL, quality of life; ARVs, antiretrovirals; RCT, randomised controlled trial; SIP, short inventory of problem.

#### Participants

Participants were between the ages of 18 years and 75 years, with sample sizes ranging from nine participants^37^ to 4847.^39^ Three studies included men only; of those, one focused on fathers with children between the ages of 8 years and 17 years,^37^ one with participants presenting with an AUD or AUDIT score ≥ 19,^40^ and one with participants attending primary healthcare centres.^41^ Two studies included women only; one with female sex workers (FSWs)^33^ and the other with women at high risk of alcohol-affected pregnancy.^35^ The remaining six studies included both men and women. One study focused on men and women on antiretroviral treatment,^38^ one focused on men and women using heroin and/or opioids,^39^ one included participants presenting for an acute visit at an emergency centre,^43^ one included participants who would continue their care at a specialist clinic for infectious diseases,^36^ and one focused on participants with methamphetamine use disorder and participants on human immunodeficiency virus (HIV) treatment.^34^

#### Design

The included studies were mainly RCTs.^33,34,35,36,38,40,41,42^ Two of the studies used qualitative research methods,^37,39^ and one study used a mixed methods approach.^43^

#### Intervention characteristics

The majority of studies targeted alcohol use.^33,34,35,36,37,38,40,41^ The three remaining studies targeted heroin use and opioids,^39^ alcohol, other drugs,^43^ and methamphetamine use.^42^

#### Intervention strategies

All interventions used either standard MI or MI-informed and/or MI-consistent interventions, which blended MI with another treatment modality. One study used standard MI.^35^ Two studies combined MI with CBT.^34,38^ One study combined MI with behavioural activation (BA),^37^ and another study combined Imaginal Desensitisation with MI (IDMI).^42^ The remaining five studies utilised MI-informed interventions, MI-informed milieux therapy,^39^ MI-informed WHO brief intervention for alcohol use,^33^ one Alcohol, Smoking and Substance Involvement Screening Test (ASSIST)-linked MI-informed session followed by two sessions of PST,^43^ positive prevention and advice plus a single brief MI-informed alcohol counselling session,^36^ and two studies used CAP (Counselling for Alcohol Problems, an MI-informed intervention).^40,41^ The number of sessions ranged from 1^40,41^ to 10,^38^ with sessions lasting from 20 min^36^ to 90 min^37^ per session.

#### Outcome measures

Seven of the 11 studies used the AUDIT as a primary outcome measure to measure the use of alcohol pre- and post-intervention.^34,35,36,37,38,40,41^ L’Engle et al.^33^ initially used the AUDIT, but later changed their measure to a behavioural interview asking specific questions about drinking behaviour. Of the four remaining studies, two used the ASSIST tool to measure the use of heroin and opioids,^39^ to measure alcohol and other drugs,^43^ and one study used the timeline follow-back method to measure the number of days on which participants had used substances within a specified timeframe and the Penn Alcohol Craving Scale (PACS) (modified for methamphetamine [MA] dependence) to measure MA use and craving.^42^

#### Impact on substance use

Overall, MI-consistent interventions were shown to improve substance use outcomes in seven of the 11 studies, with significant reductions in substance use based on outcomes measured. The study by Madhombiro et al.^38^ using a combination of MI and CBT found a significant difference in AUDIT scores at 6 months post-intervention. In their qualitative study using BA blended with MI, Giusto et al.^37^ found reduced alcohol use in their sample of alcohol-using fathers. Marks et al.^39^ could achieve their goal of a high retention rate of participants at 74% as well as high rates of harm reduction with MI-informed milieux therapy.

Van der Westhuizen et al.,^43^ utilising an intervention consisting of one ASSIST-linked MI-informed session followed by two sessions of PST, found that 59% of patients reported no alcohol use days at the 3-month follow-up, compared to 11% at screening. Moreover, the median alcohol scores had dropped significantly from the acute emergency centre (EC) visit to the 3-month follow-up.^43^ The number of drug use days in the previous 2 weeks had decreased significantly from screening to follow-up, and at the 3-month follow-up, and 75% of patients using drugs reported no drug use days in the preceding 2 weeks. Alcohol smoking and substance involvement screening test scores for patients using cannabis and methamphetamine had also decreased.^43^ Sorsdahl et al.,^42^ with the use of IDMI, found greater reductions in the frequency of MA use in the treatment group. These positive findings were consistent with treatment completers at the 3-month follow-up post-intervention.^42^

Utilising an MI-informed WHO Brief Intervention for Alcohol Use in their study with FSWs, L’Engle et al.^33^ observed a statistically significant difference in the reduction of drinking between the intervention and control groups during the 6- and 12-month follow-up visits. Specifically, the intervention group showed a more pronounced reduction in various measures related to alcohol consumption. The intervention was associated with several improvements in drinking behaviour. Participants reported drinking on fewer days in the past 30 days, engaging in fewer overall binge-drinking episodes, and having fewer binge-drinking events with both paying clients and nonpaying partners. All reductions were statistically significant (*p* < 0.0001).

The remaining five studies found less favourable outcomes. In a study by Rendall-Mkosi et al.,^35^ the only study making use of standard MI, women who received the MI intervention showed a significantly greater reduction in risk for an alcohol-exposed pregnancy (AEP) than women in the control condition. At the 12-month mark, the MI group exhibited a decline of 50.82% in the risk of an AEP, whereas the control group’s decline was at 28.12% (*p* = 0.009). Additionally, a 50% reduction in the risk of an AEP was observed in the MI group, compared to a 24.59% reduction in the control group at the 3-month follow-up (*p* = 0.004). However, the authors note that these reductions were mainly a result of improved use of contraceptives rather than reduced alcohol use.

Therefore, while demonstrating that MI may support behavioural change related to reduced risk of an AEP, the study does not provide direct evidence for MI’s effectiveness in reducing alcohol use. At the 3- and 12-month follow-ups, the MI group showed a 14.75% reduction in risky drinking compared with a 10.94% reduction in the control group; however, this difference was not statistically significant. Additionally, examining the change in median AUDIT scores from baseline to 12 months, the MI group experienced a significant reduction (a decline of 5 points) compared to that in the control group (a decline of 1.5 points as determined by Wilcoxon’s rank-sum test, *p* = 0.007).

Wandera et al.,^36^ using positive prevention and advice plus a single brief MI-informed alcohol counselling session, report mixed results. They found an overall reduction in the mean AUDIT-C score in the 6 months of follow-up: 6.4 to 3.4 (*p* < 0.0001) in the control arm and 6.8 to 3.9 (*p* = 0.001) in the intervention arm, with the greatest decline in both groups in the first 3 months post-intervention. The mean AUDIT-C score at 3 months was significantly lower in the control arm compared to that in the intervention arm (3.5 in the control group vs. 4.3 in the intervention group, *p* = 0.034); however, the mean scores were not different between groups at 6 months (3.4 in the control group vs. 3.9 in the intervention group, *p* = 0.141). The only significant finding favourable for MI was shown in a gender-stratified mixed-effects model in which the mean difference in AUDIT-C change over time was not different by treatment arm among male participants (0.38, 95% confidence interval [CI]: −0.41 to 1.17, *p* = 0.3493); but among female participants, the MI arm had greater AUDIT-C reductions compared to that in the control arm (−1.10, 95% CI: −2.19 to −0.02, *p* = 0.0457).

Madhombiro et al.,^34^ utilising a combined MI-CBT approach, found a difference in AUDIT scores over time in both the treatment and the control groups; the difference between the two groups was, however, nonsignificant. The difference in the magnitude of change between the two groups was also nonsignificant. Nadkarni et al.,^40^ using an MI-informed intervention, namely counselling for alcohol problems (CAP), found no significant difference between arms for remission at three and 12 months, and no alcohol consumption in the past 14 days at three and 12 months. For the ‘worst-case scenario’ sensitivity analysis, the difference between the two arms for proportion with remission at 3 and 12 months was not significant.^40^ Similarly, Nadkarni et al.,^41^ utilising an MI-informed intervention, CAP, found that the amount of alcohol consumed in the past 2 weeks, mean AUDIT score, and alcohol-related problems were all lower in the CAP arm compared to those in the enhanced usual care arm; however, the between-group adjusted mean differences were not statistically significant. The reductions in outcomes in participants who completed treatment compared with those who dropped out with regard to mean AUDIT scores, mean alcohol consumed in the past 2 weeks, and mean Short Inventory of Problems (SIP) score were also nonsignificant.

#### Risk of bias findings

The risk of bias was assessed for each of the included studies, wherever applicable (i.e. RCTs^33,34,35,36,38,40,41,42^). Six of the eight RCTs were considered low-risk of bias, based on the following criteria: having sufficient sequence generation, allocation concealment, blinding of participants and personnel, blinding of outcome assessors and complete outcome data reported.^33,35,36,40^ With regard to the study by Rendall-Mkosi et al.,^35^ the risk of bias associated with reporting a selective outcome was unclear as a result of the absence of information about a proposal or protocol registration number, preventing an assessment of whether all intended outcomes were reported as originally planned. The two remaining RCTs were considered high-risk as the blinding of outcome assessors was not reported for both studies.^36,40^ For one study,^40^ the blinding of participants and personnel was not reported, and for the other,^36^ based on what was reported, it was unclear whether personnel had been blinded during the intervention phase of the study. High-risk of bias with regard to these aspects of the research means one should be cautious when interpreting results across findings.

## Discussion

In this review, only one of the included studies used standard MI, not demonstrating significant reductions in substance use.^35^ The remaining studies were MI-informed, and these MI-informed interventions accounted for significant reductions in substance use and related health outcomes observed in six of the 11 studies.^33,35,37,38,39,42,43^ These findings were consistent with results found in HICs.^9,19,20,21,22,23^ The potential reasons for the remaining four studies^34,36,40,41^ yielding less favourable outcomes are various.

The study by Wandera et al.^36^ reported mixed results, with the only favourable outcomes for brief MI found among female participants. The authors proposed that one reason for their results might be that studies reporting positive outcomes in similar populations included participants whose HIV status was unknown or who were HIV negative, coming from regions with low HIV prevalence. In contrast, their study focused solely on HIV-positive participants. Furthermore, trials involving HIV-positive participants yielded similar findings of no intervention effect.^44,45,46^

In contrast to the brief single-session, MI-informed approach in this study, research using standard multi-session MI has demonstrated reductions in both the number of drinks consumed and in heavy drinking episodes. This finding suggests that multi-session MI, which aligns more closely with the original structure and phases of MI, may produce stronger behavioural outcomes than single-session adaptations do.^6,47^ Sample size may have been another contributing factor. The relatively small sample sizes in each of the four studies may not have provided sufficient statistical power to demonstrate significant treatment differences between intervention and control groups, possibly yielding imprecise effect estimates.

Regarding the interventions themselves, Nadkarni and colleagues^41^ suggested that alcohol dependence might necessitate a more intensive psychosocial treatment. The CAP intervention utilised in their study embeds the core ‘spirit’ and some techniques of MI, including client centredness, motivational enhancement, feedback, readiness to change, empathy, and collaboration. The intervention also adds substantial cognitive-behavioural and relapse-prevention content. Therefore, conclusions about MI effectiveness should be qualified as the effects of CAP reflect outcomes of an MI-informed, hybrid intervention and not necessarily what a full and/or standard MI intervention may achieve. Nadkarni et al. (2019; p.114) further proposed that additional strategies may have improved outcomes, such as potentially involving discussions on barriers to care and treatment efficacy, among others.^41^ Sileo et al.,^26^ in their meta-analysis of psychosocial interventions for alcohol use in sub-Saharan Africa, similarly argue that research on alcohol-focused interventions in this region would benefit from including more structural-level interventions such as the reduction of alcohol outlet density, enhanced alcohol regulation, and policy enforcement.

Wandera et al.^36^ found reductions in alcohol consumption as measured by AUDIT-C scores in both the intervention and the control arm of their study, implying that both interventions contained partially effective components. The comparator in their study may therefore be considered an active intervention as participants received one-on-one counselling, which differs from most other studies that use patient information leaflets only in the control arm.^48^ Prior research has shown that the simple act of participation and answering alcohol assessment questions in itself may lead to reduced reporting of drinking behaviour.^48^ The authors argue that this may have distorted the measured intervention effects and could account for the lack of difference noted in their study.^36^ Similarly, in the study by Madhombiro et al.,^34^ the comparator was the World Health Organization Mental Health Action Programme Intervention Guide, which may also be considered an active intervention and may have skewed outcomes.

### Limitations

Several limitations of the current evidence base should be acknowledged. Firstly, only one study evaluated a standard MI intervention; the remaining studies used MI-informed or blended interventions, limiting the ability to conclude about the efficacy of pure MI. Secondly, insufficient detail was provided about the specific MI techniques included, making the determination of the components that drove outcomes difficult. Thirdly, most studies did not report who delivered the interventions (e.g. lay providers vs. trained health professionals), which may affect both fidelity and generalisability. Fourthly, MI-informed or blended interventions were not consistently operationalised, limiting comparability across studies. Finally, the role of the MI component within multicomponent interventions was rarely discussed or evaluated, precluding attribution of effects specifically to MI.

Further limitations of this review stem from the variability in the methods of alcohol-use measurement in the included studies, ranging from the AUDIT, AUDIT-C, ASSIST, questionnaires, interviews, and other qualitative measures. While the search approach was systematic, only two trial registries were included (i.e. clinicaltrials.gov and the GSK Clinical Study Register [2010–2022]), which cannot ensure the identification of all interventions, as publication and language biases may have influenced the results. Moreover, certain interventions might not have been included if they did not explicitly mention MI, MI-informed, or MI-blended as a primary intervention in their methods, abstracts, keywords, or titles. One of the included studies was a pilot RCT, which may have been less robust in design and intervention content.^40^ Furthermore, upon evaluating risk of bias, two studies were identified as having a high-risk of bias^36,40^ for lacking information on the blinding of outcome assessors and participants or personnel.

## Conclusion

This review highlights that overall, MI-informed interventions demonstrated positive effects in improving substance use outcomes in the majority of the studies reviewed. Although the only study that utilised standard and/or pure MI did not demonstrate significant reductions in substance use, significant reductions were observed in several MI-informed studies. The reviewed studies employed a range of intervention strategies, including MI-blended with other interventions (such as CBT, BA, and imaginal desensitisation, and milieux therapy, among others) or using MI-informed approaches. Despite the heterogeneity, MI-informed interventions demonstrated potential effectiveness in reducing substance use. The studies were conducted in diverse settings, including primary healthcare centres, emergency centres, specialist clinics, hospitals, and Non-Governmental Organisation (NGOs). This result suggests that MI-informed interventions can be implemented effectively across various healthcare settings and different geographic locations.

The primary outcome measures varied across the studies, with the AUDIT being the most commonly used measure. While many studies reported significant reductions in substance use based on these measures, some studies showed nonsignificant differences between intervention and control groups. Future research should address the gaps highlighted above by clearly defining interventions, specifying interventionists, and evaluating the unique contribution of MI within combined programmes. Studies with larger sample sizes and more rigorous study designs are also necessary to strengthen the evidence base and address potential sources of bias. Nonetheless, MI-informed interventions can be considered a valuable approach for substance use interventions in LMICs within a range of treatment settings and available resources, complementing existing treatment modalities and promoting positive outcomes.
